# Philadelphia County Medical Society

**Published:** 1887-12

**Authors:** 


### PHILADELPHIA COUNTY MEDICAL SOCIETY.

Special Meeting, September 19, 1887.

The President, J. Solis-Cohen, M. D., in the chair.

Dr. F. W. Pavy, F. R. S., of London, addressed the society upon

### SOME MORBID CONDITIONS OF THE URINE.

I am before you to say a few words upon certain morbid conditions of the urine, and my hope is that we can compare notes of observation in the old world with observations in the new world. I shall first of all refer to albumin. Albumin in the urine with us was formerly regarded as a matter of the most serious import, but we are now beginning to recognize that a certain amount of albumin in the urine is not always of grave import. My own practice lies very largely with urinary diseases, and patients coming under my care always have the urine examined by an analyst; and I am frequently meeting with a certain amount of albumin in the urine without its presence being attended with anything that would lead me to suspect that a grave condition exists. In association with diabetes, it is not at all uncommon to find, when the patient first comes under observation, a certain amount of albumin in the urine; but when the patient is put under treatment for the diabetes, it is not infrequently found that the albumin disappears in the course of a few weeks. In these cases of albumin in the urine, we must look to the general condition of the patient to see whether or not there are other indications of the existence of grave renal disease.

There is another condition which I presume has been brought before the profession in America, in which albumin exists at a certain time of the day and not at others. I have suggested for this condition the term *cyclic albuminuria.* I have studied these cases for some years past, and I should think that they occur in this country as well as in the old world. I know that they occur in Germany, for the matter has been taken up there and followed out. This condition is observed in persons who are excitable and of a nervous disposition, and as a rule in young persons, although I have met with some cases in the middle-aged. The albumin is to be met with at one time of the day and not at others. These cases are recognized, as it were, only by accident. Sometimes the urine is examined on account of some pain in the loins, but most frequently these cases are discovered through examinations for life insurance purposes, or for some branch of the civil service, as is required by the Bank of England. So long as the individual maintains the recumbent posture, no albumin will be detected, but in one and a half or two hours after rising the maximum quantity of albumin will be found. As a rule, the quantity of albumin declines towards evening, and on going to bed it has entirely disappeared. Sometimes a trace of albumin is to be found in the afternoon and evening, but the first urine passed on rising in the morning contains no albumin whatever. These cases seem to be of no serious import. I suppose that the presence of albumin depends on the condition of the vessels. I suppose so, but certain it is that these cases are not associated with any constitutional condition. I now come across quite a large number of them.

I shall next refer to *sugar in the urine.* This is another morbid element which has varying degrees of importance. In young persons it is of the gravest import, while in elderly persons it is not so serious. In early life I have known cases to go on for years, but this is the exception; the usual duration is about two years. Ultimately they all terminate unsatisfactorily. After the middle period of life, however, sugar in the urine has a very different significance. In young subjects we have a disease which seems to be of a progressive nature. Notwithstanding whatever we may do for it, there is something which insidiously progresses in the system and leads ultimately to a fatal termination. But after the middle period of life a good deal depends upon the patient himself. Under proper management the disease may, as a rule, be held under control and the patient live for years in a satisfactory condition, but to accomplish this, rigid measures of treatment must be carried out.

One may express in a very few words, and without advocating any idea or theory, the precise nature of *diabetes mellitus*, or sugar in the urine. I should speak of it as a defective or faulty assimilative action—a faulty chemistry. The elements of the food which we take undergo chemical change in the system by which they are rendered useful. A certain group of principles entering largely into the composition of our food are the carbohydrates. These principles in diabetes do not undergo their proper chemical change and thus become eliminated from the system. What do we observe in a state of health? A person takes one of the carbohydrate group—it makes no difference which—starch, grape sugar, cane sugar, dextrine or sugar of milk—they all behave alike in the system, and it is so changed as to be rendered subservient to the requirements of the system. In a state of health we see nothing more of them. Not so in diabetes. In the diabetic these carbohydrates are no longer consumed. There is a faulty disposal of them. Received into the alimentary canal and afterward absorbed, they do not undergo their proper transformation, but pass off from the system in the form of sugar in the urine; so that I say, looking at these two conditions, a condition of health and a condition of diabetes, we may describe diabetes as a faulty disposal—a faulty transformation or assimilation of the carbohydrate elements of our food. In diabetes, in proportion as these carbohydrates are taken, so will be the amount of sugar in the urine. This I can say without any hesitation whatever.

In order to follow a case of diabetes satisfactorily, I consider that a quantitative examination of the urine should be made, and, for myself, I feel quite in the dark as to the progress of a case unless I have this quantitative examination. In my own practice, I keep an analyst, who examines the urine of patients—a night and morning specimen—and directly I get the analytical report.

I can read off exactly the condition I have to deal with. Without this I should be perfectly in the dark as regards the progress or the severity of the case. It does not do to rely on the specific gravity. I have met with a specific gravity of 1.040 without any sugar in the urine. Medical men are often concerned over the specific gravity of the urine. The patient may have been put under treatment, but still the specific gravity keeps up to 1.032 or 1.035, although the urine is free from sugar. Under these circumstances, I say to the medical practitioner: Do not concern yourself with the specific gravity. If the urine is free from sugar, the high specific gravity is a favorable sign, as showing that the kidneys are equal to good work. If the kidneys were diseased, there would be a low specific gravity. The high specific gravity may be kept up by the passage of only a limited quantity of water, and by the nitrogenous diet which the patient is taking, adding to the elimination of urea. On the other hand, a low specific gravity may sometimes be met with where there is a considerable quantity of sugar in the urine. I have met with a specific gravity of 1.009 or 1.010, and yet the urine contained a considerable quantity of sugar. These have been mixed cases of diabetes insipidus and diabetes mellitus. They are proven to be mixed cases by the fact that when the patient is put under proper dietetic treatment, the sugar disappears, but the quantity of urine keeps up. I myself do not attach so much importance to the specific gravity as is done by certain medical men.

In testing the urine for sugar, my opinion is that the copper test is by far the best. As we all know, Fehling's solution is the test generally employed, but there is this disadvantage about the Fehling's solution, it does not keep well; after being kept sometime, it throws down a precipitate without the presence of sugar. This had led to many mistakes in diagnosis. I have frequently had patients sent to me with the statement that they were suffering with diabetes mellitus, when the only trouble was that a faulty Fehling's solution had been employed. Some years ago I directed my attention to the question whether or not the ingredients of the Fehling's test could not be put together in the form of a pellet and the solution be made as required. Certain difficulties were experienced at the outset, but these have been overcome. The pellets, as now made, consist of sulphate of copper in the anhydrous form, Rochelle salt and potash. It is necessary to separate the sulphate of copper from the potash. This is done by first putting in the die the sulphate of copper, then a little Rochelle salt, next the potash, and finally more Rochelle salt. The pellet, after being made, is surrounded with waxed paper and kept in a well-stoppered bottle. If properly prepared and handled with care, the cork not being left out of the bottle, these pellets will keep indefinitely. There is this advantage about the pellet—it will never deceive you; for if it is allowed to absorb moisture it at once becomes unfit for use, the oxide of copper being thrown down in the form of a black precipitate.

In the treatment of diabetes, I attach the greatest importance to diet. I do not consider that you can get on with the management of these cases unless you exclude as far as possible those principles of which there is a faulty assimilation. If sugar appears in the urine, it must previously have existed in the blood. I know from frequent examinations that the blood contains a trace of sugar, which may be represented in figures as from 0.5 to 0.8 part per 1,000. In harmony with this condition of the blood, there has been a trace of sugar in the urine.

The ammonia-cupric test is the one I employ as a quantitative test. It consists of Fehling's solution with the addition of ammonia. With the Fehling's solution there is, on the addition of saccharine urine, a precipitate of the suboxide of copper, and, for quantitative purposes, this precipitate interferes with the determination of the exact time when complete reduction has taken place. While the presence of ammonia does not interfere with the reduction, it keeps the reduced suboxide in solution, and we get the discoloration without the formation of any precipitate whatever. The addition of ammonia gives a more intense blue color to the Fehling's solution, and this is brought by the reduction to the color of water without the formation of any precipitate. With the apparatus in perfect order, the quantitative determination can be made in two or three minutes. This test is so delicate that ordinarily, to perform it satisfactorily, you have to dilute the urine 1:20, and where it contains much sugar, 1:40. I usually determine the proportion of sugar per thousand of urine, but if it is desired to determine the number of grains per ounce, this can be done by multiplying the quantity per thousand by 0.4375.

In examining the urine of a diabetic, I generally desire that a night and morning specimen be brought. When a person lives as people ordinarily live in England—that is to say, taking a meal on rising in the morning, breakfast; a meal in the middle of the day, lunch; and a meal in the early part of the evening, dinner—the urine passed on going to bed contains considerably more sugar than the urine passed in the morning. When a person dines in the middle of the day and sups shortly before going to bed, then the conditions are reversed—there is less sugar in the night water than in the morning water. Over and over again, by the quantitative examination of the urine, I have detected errors of diet where the patients have had the greatest desire to keep to what was right. A person who has been passing only a little sugar brings a specimen which contains a large quantity of sugar. Under such circumstances, we must look for some error in the preceding meal. In one case this proved to be blanc-mange which had been made with corn flour. Blanc-mange for the diabetic should be made with cream and gelatine or isinglass. In another case a patient had been passing very little sugar, when suddenly the quantity increased. Careful questioning revealed no error in diet, until it was learned that the patient had substituted for the bran biscuits which he had been using others, said to be "just as good," which examination showed to be made of the whole meal. I refer to these cases to show the value of the quantitative analysis.

In the treatment of these cases, the exclusion of the carbohydrate elements of the food should be as complete as possible. In the case of a person in the middle period of life, I first put the patient upon a strict diet. Very often the sugar for a time lingers in the urine. It is materially diminished, but has not disappeared. I may also say that I have, in addition, recourse to opium, codeia, or morphia, for I believe that these drugs have an important influence in controlling the disease—or, to put it in other words, they restore the assimilative power. Certain it is that under the influence of these drugs and strict diet, sugar after a time disappears from the urine; and after the urine is kept free from sugar for a few months, I find that the individual has a certain amount of assimilative power over the starch of bread. I test this by giving him a couple of ounces of bread. Frequently there is no re-appearance of sugar. If there is no return after this has been continued two or three weeks, I increase the quantity to three ounces *per diem*. Then, if there is no return, to four and one-half ounces, and finally to six ounces. Then the person is in the position of a small bread-eater. I recommend persons to be content with that. They can very well give up potatoes and sugar, but to give up bread is a serious matter with many people. When a person can take six ounces of bread *per diem* he is not in an unfavorable condition. Many of these persons can continue to take this quantity of bread with no return of sugar in the urine. If, however, they go further and resume their ordinary diet, there will be a re-appearance of sugar. The urine must be taken as the guide. Treating the case in this way, my experience is that, after the middle period of life, these patients do exceedingly well.

I must apologize for the crudeness of these remarks, for I have had no time for preparation. I thank you most heartily for the attention which you have given me. I cannot close without thanking you and your countrymen for the cordial reception given to myself and my *confreres* by every one with whom we have come in contact.

Dr. James Tyson said: It is needless to say how much indebted we all feel to Dr. Pavy for his remarks and in the brief time allowed for discussion. I desire first to say that I think we, in America, have passed through much the same transition in our views as to the import of albumin in the urine as has taken place in England. The fact is thoroughly recognized that albumin may be present without any serious import; but the explanation of these curious albuminurias is still unsatisfactory. There are a certain number of them which are clearly the result of diet, and may be called alimentary albuminurias, but that all cases cannot be thus explained is shown not only by the cases alluded to by Dr. Pavy, but also in such as those where the urine on rising is free from albumin, but in which within an hour afterward the urine passed is albuminous, although no breakfast has been taken. The crucial test for the determination of the gravity of an albuminuria is the presence or absence of casts. I have found that in these harmless albuminurias tube casts are invariably absent, and where there is a constant association of albumin and tube casts, it, of course, means renal disease, no matter how slight the general symptoms may be.

With reference to sugar, we also entertain much the same views. In my hands Fehling's solution has proven the most satisfactory test, although open to the disadvantages alluded to by Dr. Pavy. Much, however, may be done to preserve it; if the bottle is kept corked and in a dark place, the solution will keep for a much longer time. I have also obtained satisfactory results in this respect from a Fehling's solution in which mannite was substituted for the tartrate of potassium and sodium. I have found that the pellets made in this country rapidly spoil.

In reference to the treatment, we largely agree. I am inclined to believe that certain sugars, and especially certain fruit sugars, are better managed by the diabetic than are others. Thus I think that a patient may eat an apple, or even an orange, without much disadvantage; whereas the use of grapes is always attended with an increase in the quantity of sugar. The same is true of milk sugar. I am satisfied that milk is a good diet for most diabetics, although, of course, it is not a cure. The dietetic treatment is, for the most, efficient; but, so far as my experience with drugs goes, I am satisfied also that the preparations of opium, and especially codeine, are the most efficient adjuncts to the dietetic treatment. They are, however, open to the objection that they produce constipation, which almost always aggravates the other symptoms of diabetes.

Dr. J. W. Holland said: The remarks of the previous peaker have reminded me of my experience with glycerine as an organic medium in Fehling's solution. This makes a solution which keeps for a considerable time, although not indefinitely. At the Jefferson College laboratory we employ two solutions of definite strength, one containing the sulphate of copper and the other Rochelle salt and the caustic potash or soda. These are mixed in equal quantities when it is desired to employ the test.

In the use of Dr. Pavy's quantitative test, I find one small objection—which can be obviated by extreme care—that is, if the solution is boiled in an ordinary capsule the ammonia will be driven off and reoxidation of the copper follows. If a flask is employed the ammonia will not escape so rapidly.

One form of pellet lately brought to my attention is the indigo-carmine pellet. This seems to keep indefinitely. It is composed of sodium carbonate and the sulphindigotate of sodium. It is a very sensitive test—even more sensitive than the Fehling's solution.

Dr. Kleen, of Karlsbad, was introduced, and said: I had, two years ago, under my care at Karlsbad, a most singular case. Mm. X., of Gothenburg, fifty years old, consulted me, telling me she was suffering with diabetes, that the illness had accidentally been discovered the year before, and that she had then visited Karlsbad (1884) and consulted Professor Seegen (who since that time had retired from practice).

Mm. X., when I saw her, presented no symptoms of diabetes or of any other illness, except some nervous debility. I gave her a considerable quantity (100 grams) of cane sugar, collected the urine for some hours, and afterward for twenty-four hours, but found no glucose in it. I did not succeed better after she had dined copiously on rice and other amylaceous food. I then pronounced her free from diabetes. I still kept the case under observation, however, and at last found, upon examining the urine two hours after a meal, which had ended with a large portion of sweet fruits (mostly pears), a small quantity of reducing substance. Both Fehling's and Nylander's (bismuth) solutions showed reactions corresponding to the one we got, with urine containing 0. 1–0. 2. per cent. of glucose. When testing the urine with polarization by the spectroscope, I found, to my utter surprise, a slight deviation *to the left.* The urine did not contain a trace of albumin. I was at a loss what to think of it, and submitted it to the test of fermentation. The same day, however, I received a letter from Professor Seegen, stating that when the patient was under his care, her urine had contained much larger quantities (as much as three per cent.) of reducing substance, and that he had found that substance to be *levulose.* At the same time Professor Seegen sent me a supply of glucose, asking me to submit the patient to a test of her toleration of that substance. I did so and found that portions of 100 grammes or more did not produce more than very small quantities of reducing substance in the urine—that substance, to judge from a very slight deviation to the left in a good instrument, being levulose.

Worm-Müller has proved that glucose, administered in large doses, passed to a certain (small) portion unchanged into the urine, even in healthy persons, while it passes to a much larger proportion into the urine of the diabetic patient. He has also found that all the different species of sugar, administered in large doses, in a certain (small) proportion pass *unchanged* into the urine even of healthy persons, while in the diabetic patient at least some portion of them passes into the urine *transformed into glucose.* He believes in this latter circumstance to have found a difference also between persons suffering from real diabetes and those whose urine, while under ordinary diet, with the common reagents, presents traces of glucose, or only now and then, under peculiar circumstances and in a passing manner, contains somewhat larger quantities of it.

This coincides with my own experience so far as it hitherto goes. Like all physicians who occupy themselves with researches in this direction, I find every year persons whose urine shows traces of sugar, and now and then—especially after strong emotions, after alcoholic excesses, or after very rich meals of rich food (but generally not after meals *exclusively* consisting of amylacea or of cane sugar)—in a passing manner, somewhat greater quantities of a substance which shows *all* the qualities of glucose. In these doubtful cases, I usually give a large quantity of cane sugar, and afterwards often find no glucose—or only slight traces of it—in the urine, till I have boiled it with mineral acid, and thus converted a portion of the cane sugar that has passed over into glucose, whereupon I will find the well-known reaction of glucose. I consider these cases to be distinct from true diabetes, though it seems to me highly probable that they indicate a tendency to that disease, which others of larger experience than myself have also noticed—for instance, Professor Frerichs.

In the present remarkable case of levulosuria, I found that some portion of cane sugar passed unchanged into the urine. It seemed to me to be of great interest to find out how Mm. X. reacted against the other species of sugar, especially against levulose, which with some reason could be expected to pass over in larger proportion than in quite normal persons. I therefore had the wish to administer a large amount of honey (which is a mixture of glucose and levulose), and also to make a test with milk sugar; but Mm. X., who had heard that she should avoid sugar above all things, and who had an interest in her own case, only partly of the same nature as my own, did not wish to be submitted to further experiments, especially as I could not promise that they would be without some momentary influence on the urine. I can, therefore, only give the above description of the case, such as already given by Professor Seegen, who first discovered it, but I am not without hope of being able in the future to give fuller information upon it.

I am decidedly of the opinion that the case is not, clinically speaking, strictly one of diabetes. If the substance had been glucose, it would have ranged among the large number of the above-named glucosurias, continuing unchanged for a great number of years in healthy or nearly healthy persons. I was rather astonished at seeing Professor Seegen state that the urine once contained as much as three per cent. of levulose, but cannot doubt the accuracy of this most excellent observer. That the quantity has diminished so very much without any dietetic treatment makes the case even more interesting than it otherwise would be. I allowed Mm. X. a tolerably good supply of starchy food, advised her only to avoid excessive use of them, and I have lately heard that the urine only now and then contains small quantities of reducing substance.

